# Volumetric analysis of cerebellar subfield structures in Alzheimer's disease revealed a different atrophy pattern using 7T ultra‐high field MRI

**DOI:** 10.1002/alz70856_100009

**Published:** 2025-12-24

**Authors:** Weiwei Zhang, Haokun Liu, Cong Zhang, Bin Jiao, Weihua Liao, Lu Shen

**Affiliations:** ^1^ Xiangya Hospital, Central South University, Changsha, Hunan, China

## Abstract

**Background:**

Emerging research highlights the cerebellum's role in cognitive and motor functions, which are commonly affected in AD patients. Studies using 3T MRI have identified structural changes in specific cerebellar subregions associated with cognitive decline and motor impairments. Ultrahigh‐field 7T MRI provides significant advantages over 3T imaging, such as enhanced contrast and higher spatial resolution, allowing for a more detailed analysis of small brain structures like cerebellar subfields. Despite its potential, 7T MRI has seen limited application in examining the cerebellum in AD patients, leaving a crucial gap in understanding whether distinct volumetric differences exist between AD patients and healthy controls. This study aims to bridge this gap by employing 7T MRI to analyze cerebellar subregion volumes in AD.

**Method:**

The study involved 30 AD patients (20 females; mean age: 61.25 ± 7.7 years) and 30 healthy controls (17 females; mean age: 55 ± 2.8 years). Imaging was performed using a Siemens 7T Terra MRI scanner equipped with a 32‐channel head coil and parallel transmission system. High‐resolution T1‐weighted images (0.7 mm isotropic) were obtained via the MP2RAGE sequence, with LayNII software used for noise removal. Preprocessing steps included intensity inhomogeneity correction through SPM12's “Segment” module and alignment to the AC‐PC plane. Accurate segmentation of cerebellar subregions was achieved using the SUITer pipeline, followed by volumetric data extraction and statistical analysis to compare groups.

**Result:**

Volumetric analysis revealed a trend toward reduced cerebellar subregion volumes in AD patients. Notably, Lobule VIIb showed a statistically significant reduction (t = ‐2.49, *p* = 0.02) compared to controls. This finding contrasts with the atrophy patterns detected by 3T MRI, which predominantly affect Crus I and Lobule VI.

**Conclusion:**

Ultrahigh‐field 7T MRI offers a promising approach for detecting specific cerebellar structural changes in AD patients. These findings underscore its potential in advancing our understanding of cerebellar involvement in AD.

